# Re-recognition of pseudogenes: From molecular to clinical applications

**DOI:** 10.7150/thno.40659

**Published:** 2020-01-01

**Authors:** Xu Chen, Lin Wan, Wei Wang, Wen-Jin Xi, An-Gang Yang, Tao Wang

**Affiliations:** 1 State Key Laboratory of Cancer Biology, Department of Immunology, Fourth Military Medical University, Xi'an, Shaanxi, 710032, P.R. China.; 2 Department of Hematology and Oncology, Children's Hospital of Soochow University, Suzhou, Jiangsu, 215025, P.R. China.; 3 Department of Medical Genetics and Developmental Biology, Fourth Military Medical University, Xi'an, Shaanxi, 710032, P.R. China.

**Keywords:** pseudogene, classification, function, diagnosis, prognosis, therapeutics

## Abstract

Pseudogenes were initially regarded as “nonfunctional” genomic elements that did not have protein-coding abilities due to several endogenous inactivating mutations. Although pseudogenes are widely expressed in prokaryotes and eukaryotes, for decades, they have been largely ignored and classified as gene “junk” or “relics”. With the widespread availability of high-throughput sequencing analysis, especially omics technologies, knowledge concerning pseudogenes has substantially increased. Pseudogenes are evolutionarily conserved and derive primarily from a mutation or retrotransposon, conferring the pseudogene with a “gene repository” role to store and expand genetic information. In contrast to previous notions, pseudogenes have a variety of functions at the DNA, RNA and protein levels for broadly participating in gene regulation to influence the development and progression of certain diseases, especially cancer. Indeed, some pseudogenes have been proven to encode proteins, strongly contradicting their “trash” identification, and have been confirmed to have tissue-specific and disease subtype-specific expression, indicating their own value in disease diagnosis. Moreover, pseudogenes have been correlated with the life expectancy of patients and exhibit great potential for future use in disease treatment, suggesting that they are promising biomarkers and therapeutic targets for clinical applications. In this review, we summarize the natural properties, functions, disease involvement and clinical value of pseudogenes. Although our knowledge of pseudogenes remains nascent, this field deserves more attention and deeper exploration.

## Introduction

Since the completion of the Human Genome Project, multiple genomic sequencing analyses have been successively accomplished in different organisms, providing numerous clues for the thorough identification of the genome, transcriptome and proteome. According to sequencing data, the entire human genome possesses approximately more than three billion bases; however, only 2% of DNA sequences encode “functional” proteins 1, and the other 98% are regarded as “trash” elements that evolved from neutral selection without coding ability. In that period, pseudogenes, along with other noncoding factors, were all categorized as “trash” sequences.

The first pseudogene was identified in 1977 when several mutations were simultaneously discovered in its DNA sequence. Due to internal mutations, the pseudogene lost its coding ability and served as a homologous gene copy as its counterpart in the genome 2. Since then, pseudogenes have been broadly identified in a series of organisms ranging from prokaryotes to eukaryotes 3. Nevertheless, because of the previous “nonfunctional” label, pseudogenes have for decades been considered as “junk DNA”, “genomic fossils” and “gene relics”; a number of strategies were even developed to focus on eliminating a pseudogene when attempting to determine its parental gene 4, 5. With the wide application of next-generation sequencing technology, pseudogenes have gradually been found to exert parental gene-dependent and parental gene-independent functions at the DNA, RNA and protein levels; these sequences are thus involved in transcriptional and posttranscriptional modulation, participating in the physiological maintenance of endogenous homeostasis and in the pathological process of disease. Notably, a small fraction of pseudogenes reportedly maintain or have regained protein-coding capacity 6, suggesting that pseudogenes also act as conspicuous elements that contributes to the transcriptome and proteome of different species.

Currently, discovered “functional” pseudogenes comprise only a small fraction of the total, whereas the majority of pseudogenes have an “unknown” status with no established identification or function. Moreover, a number of nonfunctional “dying” pseudogenes indeed are present in the genome, e.g., the ancient *O-acyltransferase-like pseudogene (ACYL3)*, increasing the complexity of pseudogene distribution 7. Pseudogenes are evolutionally conserved 8, with properties of both predisposition in unique disease subtypes 9 and tissue-specificity 10, further highlighting the potential correlation between pseudogenes and certain diseases and the necessity to study their functions and mechanisms in these diseases.

In this review, we summarize the identification, expression, evolution, biogenesis and function of pseudogenes. We also present evidence of pseudogene involvement in different diseases and the promising correlation between pseudogene and diagnosis, prognosis and therapeutics in the clinic. Finally, we discuss the current detection methods, limitations and challenges of pseudogene exploration to optimize existing protocols to increase their efficiency for further pseudogene research.

## Identification: A Long-Term Investigation

The first identification and naming of a pseudogene in human history was in 1977, when Jacq et al. 2 found a gene copy that was homologous to 5S rRNA in *Xenopus laevis*. By comparing its DNA sequence with that of 5S rRNA, they discovered a 16 base pair (bp)-deficiency and a 14 bp-mismatch condition within the 5'-terminal of this copy. In addition, its mRNA expression could barely be detected, suggesting that this gene possessed no coding capability and was considered “nonfunctional”. It was presumed that this type of aberrant gene, displaying high sequence homology to functional genes, lost its coding ability due to different mutations, such as a frameshift mutation or premature stop codon in the genome, and was termed a “pseudogene” 11.

Since then, a large number of pseudogenes have been gradually discovered from monocellular organisms to multicellular organisms and from prokaryotes to eukaryotes 12 with the aid of next-generation sequencing technologies. However, because of the high homology of pseudogene sequences to those of parental genes (termed “ancestral gene”, “cognate gene” or “counterpart”), an emerging issue faced by pseudogene analysis is how to distinguish them from their counterparts. There have long been many attempts to identify pseudogenes more accurately. First, the Ka/Ks index (rate of the nucleotide nonsynonymous to synonymous substitution) was applied as a criterion to identify pseudogenes 13 because during evolution, these sequences are under neutral selection, as opposed to positive or purifying selection. Therefore, the Ka/Ks index should be close to or equal to one 14. In fact, under the guidance of the Ka/Ks index, over 8,000 processed pseudogenes have been identified in various species 15. In this case, the Ka/Ks index serves as an initial step during pseudogene identification.

In addition to the Ka/Ks index, features of pseudogenes, such as a special category 16 and transcriptional capacity 17, became new proof for pseudogene identification, e.g., processed pseudogenes should be found in the same genome that contains their paralogs, whereas unitary pseudogenes exist alone without paralogs. Several pseudogenes were later confirmed to be transcribed, which is easily identified through RNA transcripts. Although this method may help to increase accuracy, the approach is time-consuming and not efficient because a large amount of manual work needs to be performed and pseudogenes without transcripts are difficult to identify. Therefore, this strategy is better used together with other methods. Further trials are needed to expand the search scope without decreasing accuracy.

With the rapid development of next-generation sequencing technology, strategies focusing on pseudogene identification were changed to depend on bioinformatics, which significantly promoted our recognition of pseudogenes in the whole genomes of different species. By conducting established pipelines with public databases, a large amount of comprehensive information can be acquired in a short time. However, the efficiency of this method has several limitations. 1) Because pipelines require information related to the genome, transcriptome and proteome, they are not suitable for pseudogene detection in atypical organisms. 2) Pseudogenes that are not transcribed are outside the testing range for RNA sequencing analysis. 3) Some pseudogenes only have a few nucleotides that differ from their parental genes in sequence, and an objective evaluation is needed to determine whether these differences derive from genomic mutations or sequencing errors. 4) Low expression levels and small coverage of RNA sequencing analysis are likely to result in negative results for a specific pseudogene 17, 18. Notably, despite these drawbacks, bioinformatics has become the most effective and accurate strategy for pseudogene identification.

In conclusion, pseudogene identification is a long-term investigative process from more manual works to more intelligent innovations, bringing our recognition of pseudogenes into a new era. However, more efforts should still be made to improve the breadth and precision of our current methods to help better understand pseudogenes.

## Distribution: Extremely Wide and Uneven

The distribution of pseudogenes can be classified into two perspectives, macroscopic and microscopic. From a macroscopic perspective, the distribution of pseudogenes relies on species that are different and range from monocellular organisms to multicellular organisms: monocellular organisms have few or no pseudogenes with exclusive effects, whereas multicellular organisms, including prokaryotes and eukaryotes, possess many more pseudogenes 3. Almost 11,000 pseudogenes have been identified in the complete human genome 19, and more than two-thirds (over 8,000) have been verified as processed pseudogenes 15.

From a microscopic perspective, only 10% of the genes in the entire human genome can be detected with at least one pseudogene counterpart. Moreover, the distribution of pseudogenes per coding gene is markedly uneven 20, 21. Notably, pseudogenes are frequently located in regions undergoing DNA duplication, deletion or chromosomal rearrangement 22, which may give rise to more mutations in those sequences. In addition to the global distribution of pseudogenes, the total amount of transcribed pseudogenes varies widely, ranging from 6% 23 to 20% 24. Compared with their parental genes, the RNA transcripts of pseudogenes also change significantly in abundance because decreased levels are found for the majority of pseudogene transcripts 25. Nonetheless, for some examples, such as pseudogenes of *POU class 5 homeobox 1 (OCT4)*, the levels are almost equal to or even increased 26, further indicating the uneven distribution of pseudogenes at the molecular level.

Taken together, these details highlight the extremely wide and uneven distribution of pseudogenes within the genome at macroscopic and microscopic levels, suggesting their intrinsic diversity and complexity in genomes.

## Expression: A Spatiotemporal and Unique Pattern

The expression pattern of a pseudogene shows a strongly spatiotemporal property compared with that of its parental gene, and these expression patterns appear to occur in two completely opposite phases. In fact, most pseudogenes are expressed in parallel with their parental genes, e.g., loss of *phosphatase and tensin homolog pseudogene 1 (PTENP1)*, a processed pseudogene of *PTEN* at chromosome 9p13.3, can lead to a remarkable reduction in the level of *PTEN* 27. Both the *PTEN* and *PTENP1* loci may be deleted in melanoma 28, suggesting a positive spatiotemporal correlation between the parental gene and its pseudogene. However, several pseudogenes exhibit an expression pattern that is entirely different from that of their parental genes, e.g., the *5-hydroxytryptamine receptor 7 (HTR7)* pseudogene can be detected in the liver and kidney, whereas its counterpart *HTR7* is exclusively present in the central nervous system (CNS). Additionally, RNA transcripts of *secretory blood group 1, pseudogene (SEC1P)* have been found in all tumor cell lines detected, but those of its parental gene *fucosyltransferase 2 (FUT2)* were not found in six leukemia cell lines despite the same chromosomal location and almost 70% homology, as supported by evidence from Koda et al. 29. Therefore, spatiotemporal expression specificity is probably the reason that pseudogenes can function in a parental gene-dependent or parental gene-independent manner.

In addition to a spatiotemporal expression pattern that is different from that of its parental gene, a pseudogene also shows a unique expression profile in different specimens and under various conditions. First, pseudogenes frequently display a tissue-specific expression profile in different organs, tissues, and even blood; for example, *SUMO1P*, a pseudogene of *small ubiquitin like modifier 1 (SUMO1)*, is upregulated in gastric cancer (GC) tissues compared with benign gastric disease tissues 10, and expression of *integrator complex subunit 6 pseudogene 1 (INTS6P1)* in the plasma of hepatocellular carcinoma (HCC) patients is significantly decreased compared with the plasma of non-HCC patients 30. Pseudogenes also appear to be expressed in a specific disease subtype. For instance, Kalyana-Sundaram et al. performed an RNA-seq analysis on samples from 13 cancers and their corresponding normal tissues and found 218 pseudogenes and 40 pseudogenes that were only present in the cancer samples and a single cancer subtype, respectively 31. Similarly, the pseudogene *Nanog homeobox retrogene P8 (NANOGP8)* is aberrantly expressed in cancer cell lines, though its counterpart *NANOG* is not 32. Furthermore, different physiological or pathological conditions may lead to alterations in pseudogene expression, such as cell differentiation 33, diabetes 34, asthma 35 and cancer 36, 37. Moreover, single-nucleotide polymorphisms (SNPs) can occur in pseudogene sequences to induce variants, such as alleles of *poly(ADP-ribose) polymerase (PADPRP)-processed pseudogene* 38, *E2F transcription factor 3 pseudogene 1 (E2F3P1)* 39 and *OCT4-pg1* 40. Finally, epigenetic modifications, such as DNA methylation, are involved in modulating pseudogene expression, e.g., the promoter region of *PTENP1* in GC cells is dramatically enriched with DNA methylation, leading to an epigenetic silencing effect 41. In conclusion, a pseudogene has its own expression pattern, which is different from that of the parental gene, in some disease conditions, serving as a potential biomarker in clinical applications.

## Evolution: “Molecular Fossil” and “Gene Repository”

The identification of pseudogenes has revealed an interesting phenomenon in which pseudogenes are highly homologous to their parental genes because of their origin, strongly indicating their evolutionary conservation. In addition, the ratio of nucleotide nonsynonymous to synonymous (Ka/Ks) mutations of a pseudogene is close to or equal to one, which is relatively high, suggesting that despite the mutations involved, the pseudogene is under an evolutionary constraint 8. Moreover, with the preservation of a specific sequence, a pseudogene has its own identity when evaluating genetic relationships and evolutionary distances between species, acting as a “molecular fossil” or “gene relic” in the genome 42. For instance, Marques et al. 43 found that a total of 48 pseudogenes are conserved in various specimens, including humans, mice, rats and dogs. Another recent study identified 68 pseudogenes that are conserved in humans and two other mammals 44, indicating high evolutionary conservation of the pseudogene in primates.

In fact, pseudogenes are under neutral selection pressure to be maintained in the human genome 15, and they gradually develop functions that are similar to or even greater than those of their counterparts 45, functioning as a “gene repository” to store and expand genetic information. Furthermore, the number of pseudogenes in the genomes of multicellular organisms is much higher than that in the genomes of monocellular organisms, and monocellular organisms are capable of excluding genes that have become pseudogenes, further indicating the “gene repository” role of pseudogenes in higher organisms 3.

Nevertheless, despite some current evidence proving the conserved evolution of pseudogenes, more efforts should be made to increase the proof and to elucidate the underlying mechanisms.

## Biogenesis and Classification

### Biogenesis: A pseudogene is regarded as a product and a reservoir of gene mutations

Due to the duplication and transcriptional properties of the human genome, more than one product of a gene is produced, which significantly promotes genetic information heritance but lays a foundation for pseudogene biogenesis. Pseudogenes are primarily derived from two events. 1) Mutation: a gene that is newly generated during DNA duplication or multiple mutations (such as insertion, deletion, frameshift, premature stop codon, and splicing error in the coding or regulatory regions) can give rise to loss of its function, especially the protein-coding property, and can transfer it to a pseudogene 46. Similarly, for the original functional gene, the accumulation of mutations in certain domains can also convert it to a “nonfunctional” pseudogene 25. 2) Retrotransposon: reversely transcribed cDNA may randomly reintegrate into the genome by forming an inappropriate locus or mutation, leading to the biogenesis of a functionally insufficient pseudogene 17. Notably, based on the abovementioned evidence, the biogenesis of pseudogenes is more likely to proceed during high-synthesis and high-metabolism DNA events, which provide more opportunities for mutations; this is supported by evidence that pseudogenes may be present at a higher rate in reproductive cells than in somatic cells 47. Therefore, pseudogenes serve as an outcome and the simultaneous storage of gene mutations in the human genome.

In theory, any sequence in the genome can give rise to a pseudogene because the key trigger is a mutation that frequently and inevitably occurs. However, some elements are likely to affect pseudogene biogenesis. 1) Type of nucleotide: Pseudogene biogenesis is less common in regions enriched with GC nucleotides, which is probably due to their negative effects on the accumulation of mutations 48. 2) Length of the gene: A different coding gene length tends to produce a different subtype of pseudogene, e.g., a processed pseudogene is generally found in a short coding gene 49. 3) Gene condition: As mentioned above, certain genes that are frequently involved in a high duplication status can increase the possibility for mutations, such as highly expressed genes in cell division and metabolism 50. 4) Pseudogene: Evidence shows that the parental gene is not the only source of a pseudogene, which can also derive from another pseudogene 51. These findings not only reveal the diversity of pseudogene biogenesis but also provide novel approaches for pseudogene identification.

### Classification: A pseudogene is categorized via a distinct biogenesis process

**Figure 1:**
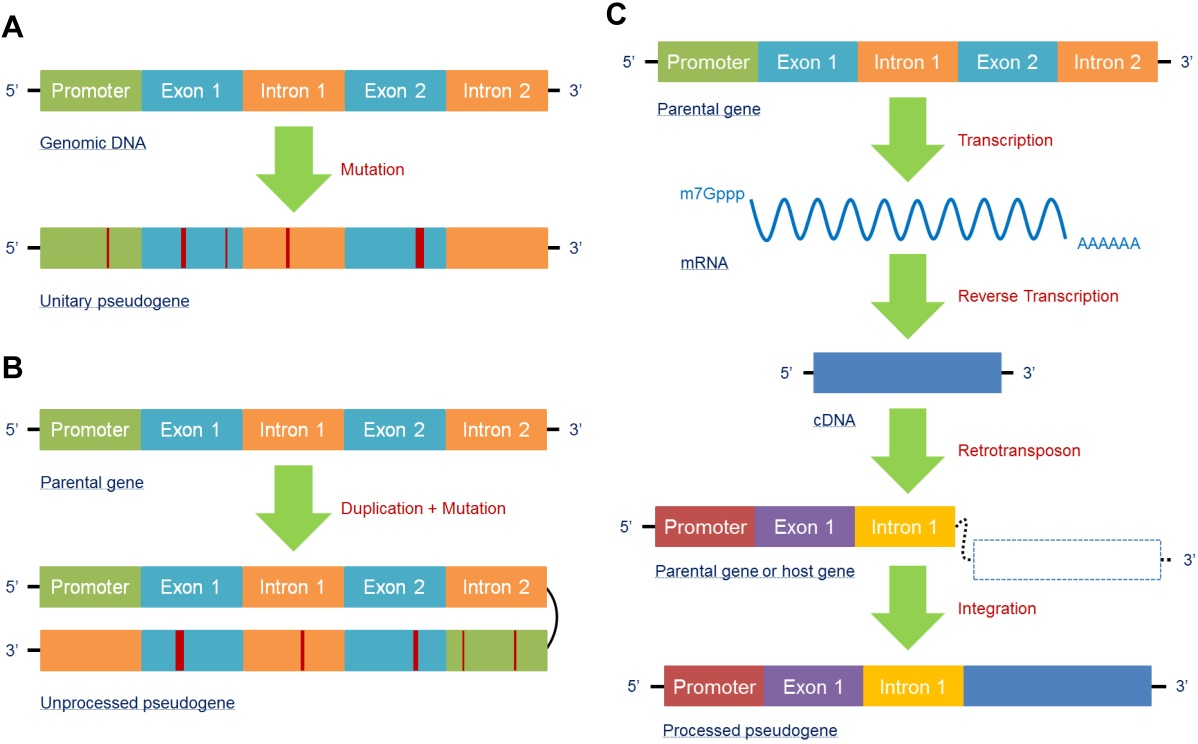
Pseudogenes are mainly generated in three forms. **(A)** The unitary pseudogene is derived from a coding gene with several mutations involved, leading to loss of its transcription and translation capacities, with have no fully functional counterpart in the same genome. **(B)** Due to unfaithful duplication, the duplicated gene generates a mutated gene copy that eventually becomes an unprocessed pseudogene; the original gene copy is fully functional. **(C)** A processed pseudogene derives from an mRNA that has been reverse transcribed into a cDNA and then synthesized into a host gene or parental gene via retrotransposon. Processed pseudogenes can be found far from their counterparts or on different chromosomes.

In accordance with the unique biogenesis mechanism, pseudogenes can generally be classified into three types: unitary pseudogenes, unprocessed pseudogenes, and processed pseudogenes. A unitary pseudogene, as its name suggests, is derived from a single coding gene copy with a few mutations that restrain transcription or translation. Hence, there is no fully functional genomic counterpart for the unitary pseudogene in the same genome, though orthologs can be found in related species 16 (**Figure 1A**). An unprocessed pseudogene, also known as a duplicated pseudogene, is the product of aberrant DNA duplication with mutation. Although it is located in the same region of a chromosome and contains the introns with flanking sequences of its counterpart, its ability to be transcribed or encode proteins is lost due to mutations in coding or regulatory sequences; in contrast, its counterpart retains its original functions 52 (**Figure 1B**). A processed pseudogene is different from the above two types because its main mechanism of formation is the retrotransposon of mRNA transcripts. As a result, processed pseudogenes may contain poly(A) tails without introns and regulatory sequences and integrate randomly into the genome; thus, they are more likely to be found in a new location far from their counterparts or on different chromosomes 53. Additionally, as retrotransposon is not a high-fidelity process, mutations may occur within the processed pseudogene to suppress its function 54. Moreover, transcription of a processed pseudogene relies on the regulatory elements of its host gene because it lacks a promoter, which is different from its parental gene 24 (**Figure 1C**). In fact, because of the specific biogenesis mechanisms and structures, the abovementioned three subtypes of pseudogenes can display a variety of functions that help them participate in the regulation of gene networks and diseases.

## Functions

At different levels, a pseudogene serves a variety of functions other than that of a “nonfunctional” gene “trash” or “relic”. For example, at the DNA level, pseudogenes can impact their parental gene or host gene sequences by random insertion or DNA sequence exchange, thus further influencing their structures and functions. At the RNA level, RNA transcripts of pseudogenes can function as antisense RNAs, small interfering RNAs (siRNAs) and competing endogenous RNAs (ceRNAs) to regulate target gene expression at the posttranscriptional level. At the protein level, pseudogenes may be able to encode a protein or peptide to act as a “functional” gene involved in a gene regulation network. Therefore, pseudogenes are important because they thoroughly influence the human genome under different conditions, especially in diseases.

### Function of pseudogenes at the DNA level

#### Random insertion

The DNA sequence of a pseudogene is able to randomly insert into the host gene to exert different effects, which mainly depend on the specific region of insertion. The insertion forms and effects are as follows.

1) Epigenetic silencing: By inserting into the upstream regulatory regions, particularly a promoter, a pseudogene may destroy its “landing site” and prevent host gene transcription, e.g., pseudogene *protein tyrosine phosphatase non-receptor type 12 (PTPN12)* inserts into the promoter region of *MAX dimerization protein MGA (MGA)*, which acts as a potential lung cancer suppressor, to inactivate its expression and promote a malignant phenotype in NCI-H2009 cells 31. Given that mutation occurring in antitumor genes is a key trigger of tumorigenesis, pseudogene-induced epigenetic silencing of antitumor genes provides a new form of mutation during tumor development.

2) Initiating transcription: When a pseudogene insertion site occurs in the intron or 3'-untranslated region (3'-UTR) of a host gene, the pseudogene is capable of using the transcriptional launch of the host gene to help trigger its own expression; in contrast, a pseudogene is unlikely to be expressed when inserting into an intergenic region. Notably, when a pseudogene insertion site is in the 3'-UTR of a host gene, all 3'-UTR-induced posttranscriptional regulation is lost 55. In fact, this feature may promote the detection of some pseudogenes that cannot be transcribed in their original gene loci.

3) Genetic fusion: A processed pseudogene, which is inserted into a more downstream intron site of a host gene, tends to be cotranscribed with its own host gene, giving rise to a fusion RNA transcript that is partially derived from the pseudogene and partially derived from the host gene. For example, Koda et al. discovered a fusion gene of *FUT2* and its pseudogene *SEC1P*, which is formed by an unequal crossing-over. The fusion gene contains the 5' region of *SEC1P*, indicating that it shares the same promoter region with *SEC1P* 29. Because *SEC1P* can be detected in multiple cancer cell lines, expression of the *FUT2* and *SEC1P* fusion is expected in tumors *in vivo*.

4) Mutagenesis: Transcription of the host gene can be abrogated when the insertion site of a pseudogene is within an exon, similar to an insertion mutation in a coding sequence 56. This situation leads to functional loss of normal genes, especially those that are significant for homeostasis, which would result in microenvironment disruption and/or disease.

**Figure 2:**
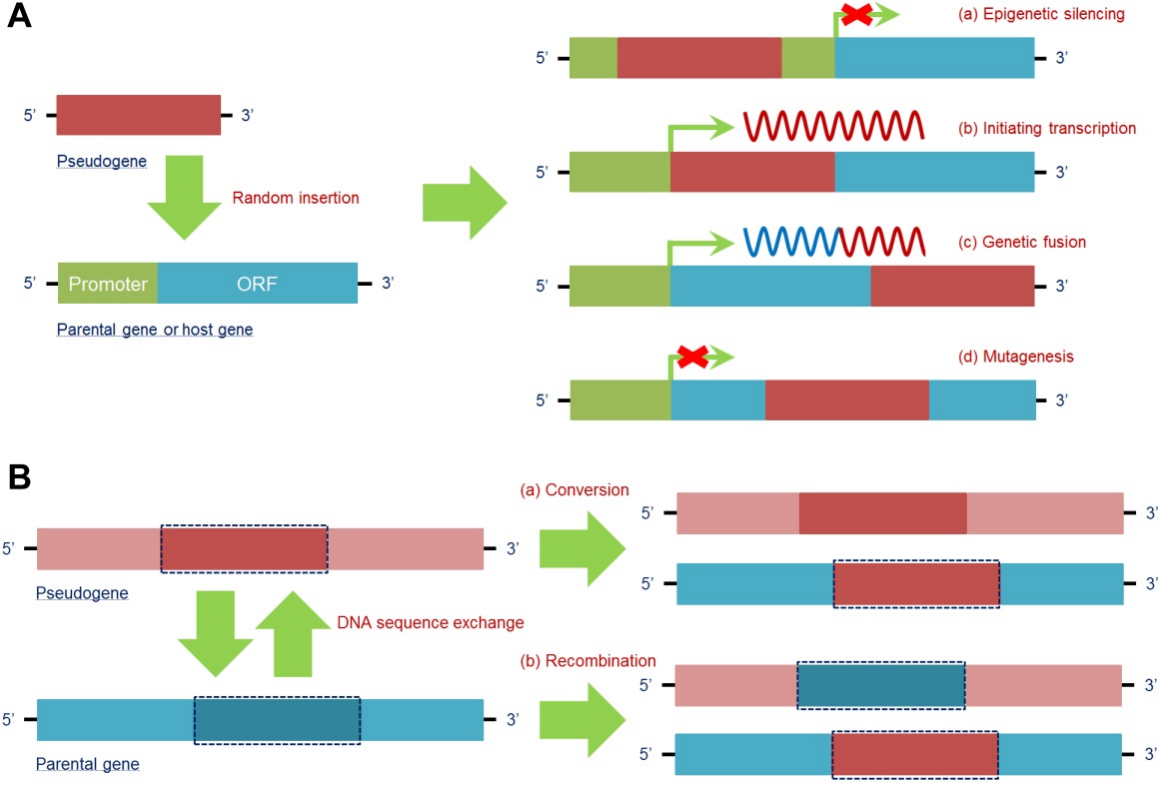
Pseudogenes have a series of regulatory effects at the DNA level. **(A)** DNA of the pseudogene can be randomly inserted into the parental gene or other host genes to regulate their transcription. By insertion into the promoter region of a target gene, a pseudogene is able to epigenetically silence its expression (a). In addition, pseudogenes can utilize the transcriptional mechanism of host genes to achieve their own transcription (b). Moreover, when the insertion site of a pseudogene is in a more downstream intron site of the host gene, a fusion gene is formed, and a chimeric RNA transcript is then produced (c). In fact, pseudogene insertion occurring in the coding region of a target gene can lead to mutagenesis and simultaneously loss of its own function (d). **(B)** DNA of the parental gene can also be influenced by pseudogenes via sequence exchange due to the high similarity of the sequences. Parental gene DNA can be substituted in a conversion with (a) or exchanged in a homologous recombination with (b) specific pseudogene DNA, thus finally affecting the function of the parental gene at the DNA level. ***Abbreviations*:** ORF: open reading frame.

Therefore, pseudogene insertion can be regarded as a new area for recognizing disease mechanisms, especially for tumors because insertion frequently occurs (**Figure 2A**).

#### DNA sequence exchange

In addition to random insertion into a host gene, a pseudogene can perform a parental gene-dependent function by exchanging DNA sequences with the gene. The main pattern of this function exhibits two types. 1) Conversion: The DNA sequence in the parental gene can be substituted by the homologous sequence in the pseudogene. After this replacement, the newly substituted section and the rest of the sequence in the host gene become identical. For instance, the hybrid alleles *PMS1 homolog 2, mismatch repair system component (PMS2)* can carry sequences from its pseudogene, *PMS2 C-terminal like pseudogene (PMS2CL),* in exons 13-15, tracing back to an intrachromosomal recombination that possibly modulated cancer susceptibility in carriers 57. Furthermore, conversion between pseudogenes and their parental genes provides a great opportunity for activation of oncogenes or inactivation of oncosuppressor genes 58. 2) Recombination: Homologous sequences between a pseudogene and its counterpart can be exchanged, disrupting the original function of the parental gene. For instance, intron 2 of *BRCA1 DNA repair associated (BRCA1)* and intron 2 of its pseudogene *PsiBRCA1* can be exchanged by recombination, transferring *BRCA1* into a “nonfunctional” gene without a tumor suppressive effect 59, which constitutes a new mechanism for inactivating antitumor genes. In this case, DNA sequence exchange between the pseudogene and its counterpart provides a chance for disease occurrence (**Figure 2B**).

### Function of pseudogenes at the RNA level

#### Transcription as an antisense transcript

**Figure 3:**
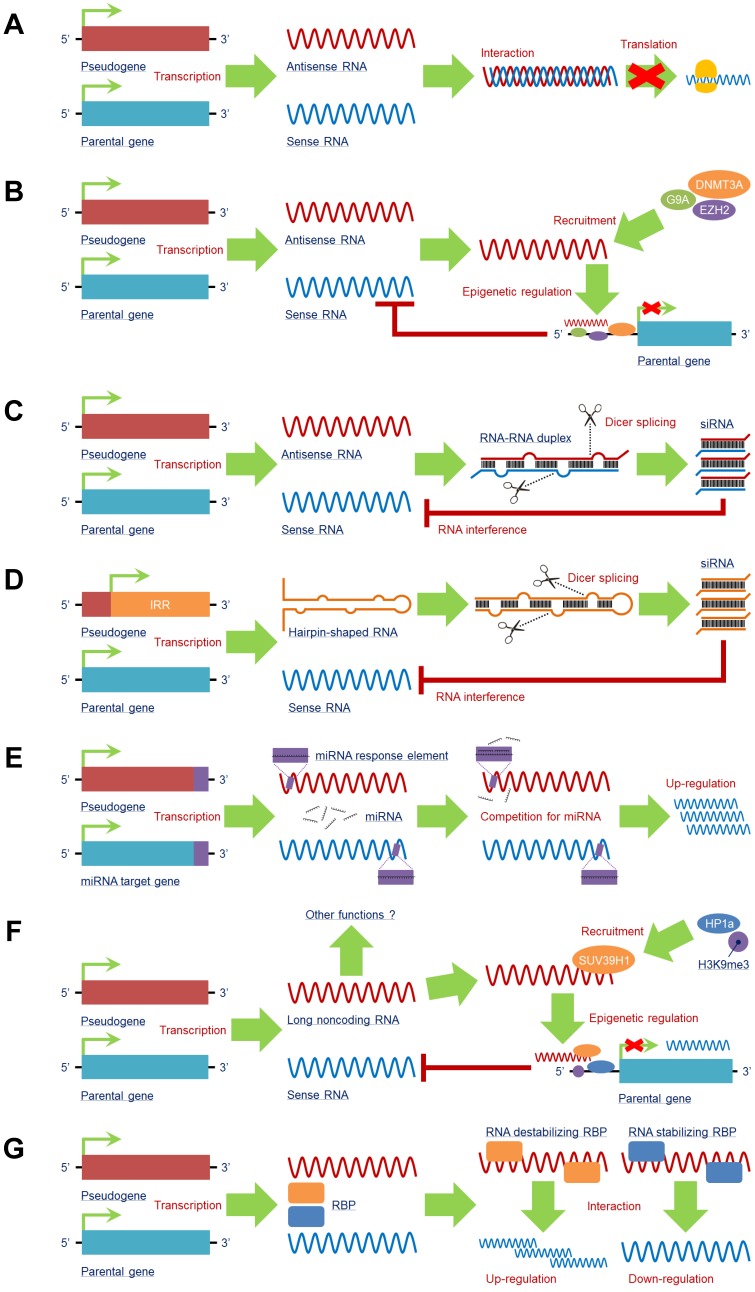
Pseudogene RNA exists in various forms and plays a vital role in target gene expression. A pseudogene can be transcribed into antisense RNA that **(A)** interacts with or **(B)** recruits multiple negative epigenetic regulators, such as G9A, DNMT3A, and EZH2, to the promoter region of the parental gene to induce an inhibitory effect on its transcription. Some pseudogenes can produce endogenous siRNAs by **(C)** interacting with sense RNA to form a double-stranded RNA-RNA duplex or **(D)** being transcribed from the inverted repeat region. Both of these products may undergo Dicer splicing to produce siRNAs, which mediate an RNA interference effect to reduce sense RNA. **(E)** By containing similar miRNA response elements (MREs) with the miRNA target gene, including the parental gene, pseudogene RNA is capable of competing for miRNAs by serving as a miRNA decoy to enhance expression of miRNA target genes at the posttranscriptional level. **(F)** Pseudogenes can also generate long noncoding RNAs (lncRNAs) to trigger epigenetic regulations of parental genes; other functions of pseudogene-derived lncRNAs still need to be investigated. **(G)** Pseudogene RNA is able to compete for RNA-binding proteins (RBPs) with the RNA transcripts of the parental gene. The comprehensive effect on the transcription of the parental gene mainly relies on the subtype of RBP: RNA-stabilizing RBPs and RNA-destabilizing RBPs. ***Abbreviations*:** DNMT3A: DNA methyltransferase 3 alpha; EZH2: enhancer of zeste 2 polycomb repressive complex 2 subunit; G9A: euchromatic histone lysine methyltransferase 2; H3K9me3: histone trimethylated at lysine 9; HP1a: Heterochromatin Protein 1A; IRR: inverted repeat region; RBP: RNA binding protein; SUV39H1: suppressor of variegation 3-9 homolog 1.

A pseudogene can produce antisense RNA transcripts that directly interact with the mRNA of its counterpart, generating a double-stranded RNA-RNA duplex to restrain translation of the counterpart. A canonical example of this function is the pseudogene of *neuronal nitric oxide synthase* (*nNOS*), which is a natural antisense regulator and transcribed with a significant antisense region to *nNOS*, resulting in the formation of a double-stranded RNA-RNA duplex and a decline in nNOS protein synthesis. Both the *nNOS* and pseudo*NOS* transcripts are present in the same neuron, and the activity of the nNOS enzyme is substantially suppressed 60, suggesting that the pseudogene-mediated antisense mechanism can regulate the translation of some neuron-dependent genes and that simultaneously transcribed pseudogenes are a potential source of a new class of regulatory genes in the central nervous system (**Figure 3A**).

Intriguingly, potential crosstalk exists between the pseudogene antisense RNA transcript and epigenetic regulation, e.g., the antisense RNA alpha isoform of *PTENP1* can recruit DNA methyltransferase 3 alpha (DNMT3A) and enhancer of zeste 2 polycomb repressive complex 2 subunit (EZH2) to the promoter region of *PTEN* to suppress its transcription, whereas the beta isoform of *PTENP1* antisense RNA still performs the traditional function 61. *OCT4-pg5* is able to produce to an antisense RNA that enhances enrichment of histone trimethylated at lysine 27 (H3K27me3) to repress *OCT4* transcription by recruiting EZH2 and euchromatic histone lysine methyltransferase 2 (G9A) to its promoter region 62 (**Figure 3B**), indicating that pseudogenes may have both transcriptional and postregulatory effects on parental genes.

#### Processing into siRNAs

Several pseudogenes are capable of generating endogenous siRNAs. There are two major mechanisms that pseudogenes rely on for processing into siRNAs. One is from RNA-RNA duplexes formed by sense mRNA and antisense RNA transcript from counterpart pseudogenes (**Figure 3C**); the other is from hairpin-shaped RNA generated by the inverted repeat region of the pseudogene (**Figure 3D**). Both products can be sliced into siRNAs by Dicer. The single-stranded siRNAs are incorporated into an RNA-induced silencing complex to initiate the RNA interference (RNAi) process to regulate the counterpart gene 63.

*Protein phosphatase, Mg^2+^/Mn^2+^-dependent 1K pseudogene (PPM1KP)*, a partially retro-transcribed pseudogene with an inverted repeat region, can fold into a hairpin-shaped structure to produce two endogenous specific siRNAs that negatively regulate its cognate genes *PPM1K* and *NIMA-related kinase 8 (NEK8)* 64, leading to alterations in hepatocellular cell mitochondrial activation and proliferation. This further illustrates double pseudogene functions as both parental gene dependent and independent.

In fact, multiple pseudogenes have been found to create siRNAs to inhibit the functions of their counterpart via the RNAi mechanism, in humans 65 and also in mice 66, insects 67, and plants 68, 69, suggesting that siRNAs derived from pseudogenes are not exclusive to humans and may be discovered in other species not yet been reported.

#### Competition for miRNAs

MicroRNAs (miRNAs) comprise a cluster of small noncoding RNAs that function as negative regulators of target genes by interacting with miRNA response elements (MREs) in the mRNA 3'-UTRs at the posttranscriptional level. Theoretically, any RNA possessing MREs can sponge miRNAs, which are also categorized as ceRNAs 70, including pseudogene RNA transcripts. In fact, pseudogene RNA transcripts may contain several MREs that are the same as those in their counterparts, leading to direct competition for miRNAs and allowing pseudogenes to modulate their counterparts. By sharing similar MREs, target genes other than their counterparts can also be regulated by pseudogenes in a ceRNA manner 18, 71 (**Figure 3E**).

Processed pseudogenes *HMGA1P6* and *HMGA1P7*, located at 13q12.12 and 6q23.2, share high sequence homology with their parental gene *high mobility group AT-hook 1 (HMGA1)* in the 3'-UTR. Within this region is a perfect match with the conserved seed sequences of *HMGA1*-targeting miRNAs, e.g., miR-15, miR-16, miR-214 and miR-761. In addition, *HMGA1P6* and *HMGA1P7* show a similar expression pattern with *HMGA1* due to their competition as ceRNAs for the endogenous binding sites of *HMGA1*-targeting miRNAs; they therefore function as oncogenic regulators of their parental gene *HMGA1* in human anaplastic thyroid carcinomas 72.

Similarly, *BRAFP1*, a pseudogene of *B-Raf proto-oncogene, serine/threonine kinase (BRAF)*, is a genomic gain and aberrantly expressed in various human cancers. *BRAFP1* serves as a miRNA decoy that sponges miR-30a, miR-182, miR-590 and miR-876 to regulate expression of *BRAF* and to activate the mitogen-activated protein kinase (MAPK) signaling pathway, inducing lymphoma *in vivo* 73. *OCT4-pg4*, which is abnormally activated in HCC, can restrain the inhibitory effects of miR-145 on *OCT4* by competing for the binding site with miR-145, thus increasing the expression level of *OCT4* to promote HCC cell growth and tumorigenesis *in vitro* and *in vivo* 74.

In summary, pseudogenes can protect the mRNAs of the parental genes or of genes possessing the same MREs from degradation by competitively interacting with suppressive miRNAs, indicating a parallel expression pattern between the pseudogene and the miRNA target gene. Moreover, these findings add a new layer of posttranscriptional regulation of target genes and shed novel light on the treatment of certain diseases.

#### Production of lncRNAs

In addition to RNA transcripts of antisense RNAs and siRNAs, pseudogenes can produce long noncoding RNAs (lncRNAs), as supported by data from next-generation sequencing analysis 31. LncRNAs are characterized as a class of ncRNAs with sequence lengths over 200 nucleotides that exert a series of functions in biological processes, such as transcription, translation and epigenetic modification 75, 76. By producing lncRNAs, pseudogenes can modulate gene expression in a lncRNA-like manner. However, studies in this field are still in their infancy; indeed, only a small number of the pseudogene-derived lncRNAs have been found 77, and very little is known about their functions. As an example, the lncRNA of murine *Oct4P4* can bind to suppressor of variegation 3-9 homolog 1 (SUV39H1) HMTase to form a complex that recruits histone trimethylated at lysine 9 (H3K9me3) and Heterochromatin Protein 1A (HP1a) to the *Oct4* promoter region, leading to a silencing effect on *Oct4* expression 78. This is a relatively clear mechanism. *SUMO1P3* is a pseudogene of *SUMO* that can generate lncRNAs, though the detailed functions of these *SUMO1P3*-derived lncRNAs remain unclear 10. The clinical significance of these pseudogene-derived lncRNAs is also largely unknown (**Figure 3F**).

#### Interaction with RNA-binding proteins (RBPs)

RBPs, which play two entirely opposite roles in mRNA expression as stabilizing or destabilizing factors, serve as critical regulators at the posttranscriptional level. By functioning as stabilizing factors, RBPs help protect mRNAs from degradation; as destabilizing factors, RBPs alleviate the stability of target mRNAs. As a pseudogene presents high sequence similarity to its counterpart, the RNA transcripts of a pseudogene can both interact with RBPs and compete with the counterpart for binding sites in RBPs. Therefore, according to the roles of RBPs, pseudogenes function as positive or negative regulators to help stabilize or destabilize target mRNAs when their RBPs are bound to the pseudogene 70, 79 (**Figure 3G**).

Coexpression of *myosin light chain kinase pseudogene 1 (MYLKP1)*, a pseudogene of *MYLK* with *smMLCK*, an encoded isoform of *MYLK*, promotes reduced *smMLCK* mRNA stability, suggesting potential competition between the RNA transcripts of a pseudogene and its counterpart for RNA-stabilizing RBPs 80. This may be the reason that *MYLKP1* negatively regulates its parental gene *MYLK* expression, is positively correlated with multiple tumors and increases tumor cell proliferation. Overall, there is little evidence for positive effects on target gene expression of interaction of a pseudogene with RBPs.

### Function of pseudogenes at the protein level

#### Encoding a protein or peptide

According to its identification, a pseudogene is labeled with “pseudo” due to its deficiency in protein coding. Nevertheless, some fully processed pseudogenes maintain or regain this capability, in contrast to the majority of identified pseudogenes.

The first known protein-coding pseudogene was *phosphoglycerate mutase 3, pseudogene (PGAM3)*, which was formed by the *PGAM1* retrotransposon. The protein-coding ability of *PGAM3* had not been verified until Betran et al. 6 identified it through polymorphism and expression data. Another classic example of a protein-producing pseudogene is *OCT4-pg1*, which contains an entire open reading frame (ORF) that produces a protein that is localized to the nucleus and does not have similar activities as its parental gene *OCT4* 81.

**Figure 4:**
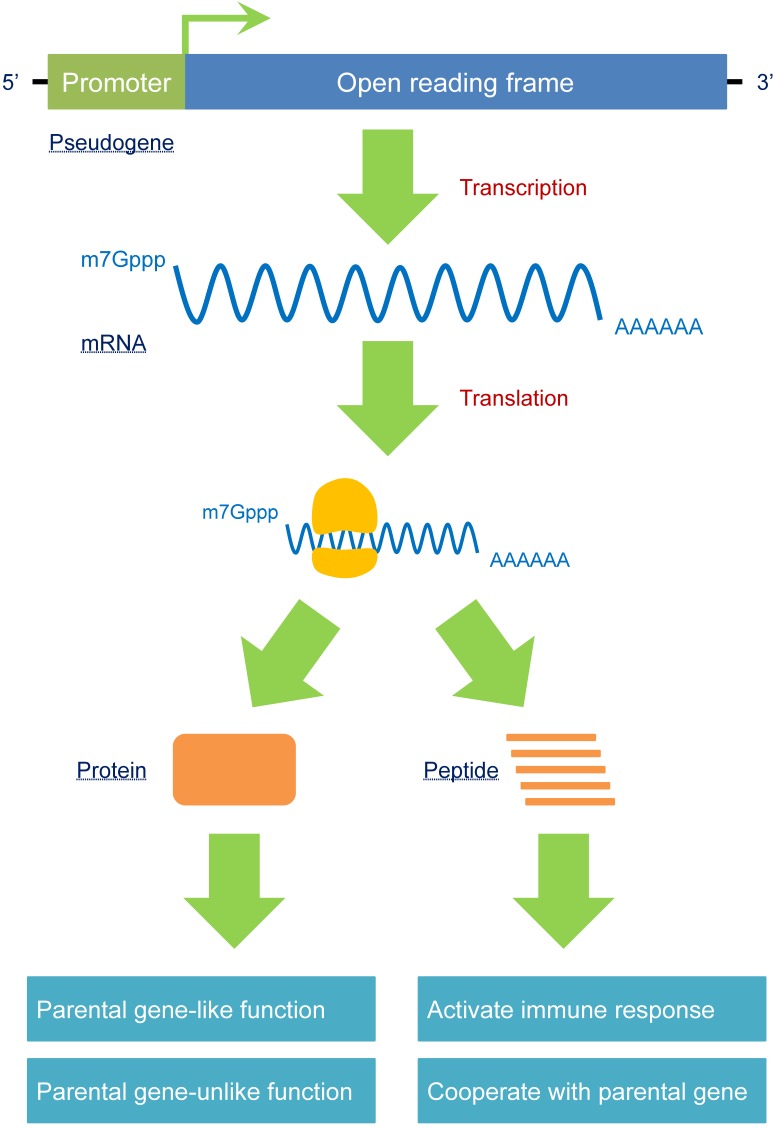
A pseudogene should no longer be treated as a “nonfunctional” element. The critical criterion for judging whether a gene is “functional” or not is predominantly according to its encoding-protein ability. In fact, some pseudogenes harbor a complete open reading frame (ORF) to produce mRNAs. Therefore, these pseudogenes can produce proteins that exert parental gene-like or parental gene-unlike functions. In addition, a small number of pseudogenes can be transcribed as fragments of entire mRNAs, generating different peptides that can induce immune responses or cooperate with parental genes. Because pseudogenes have the potential to produce proteins, as opposed to the traditional opinions that pseudogenes are “nonfunctional”, a reasonable nomenclature is required to reidentify these special types of sequences.

In addition to the entire functional protein encoded by an entire ORF, a pseudogene may generate a peptide without full function because of mutations, especially if the pseudogene encodes a premature stop codon; e.g., *BRAFP1*, located on chromosome Xq13, is interrupted by many stop codons that prevent it from being translated into a fully functional protein. The longest peptide, with a total of 244 amino acids, has a sequence that is highly homologous to the CR1 domain of and cooperates with the BRAF protein to promote MAPK signaling 82, leading to enhanced tumorigenesis in thyroid tumors. Notably, pseudogene-derived proteins or peptides can be treated as “antigens” by the human immune system to induce immune responses 83, which may lead to novel and promising biomarkers or therapeutic targets for certain diseases (**Figure 4**).

## Disease Involvement

Due to the incredible development of next-generation sequencing technology, a large number of pseudogenes have gradually been discovered. Despite some “dying” pseudogenes that have been demonstrated to be nonfunctional, there is sufficient evidence to confirm that pseudogenes harbor various functions at the DNA, RNA, and protein levels, participating in the modulation of target gene expression, particularly their parental genes. Therefore, these molecules are involved in the development and progression of certain diseases, especially cancer. In addition, pseudogenes present their own expression patterns in different species, with a connection to disease diagnosis and prognosis (**Table 1**). Moreover, many studies have attempted to overexpress or inhibit specific pseudogenes to detect their effects on different diseases *in vitro* and *in vivo* (**Table 2**), providing clues for clinical reference to formulate reasonable treatments. Although pseudogene studies are only beginning to be initiated, they have revealed broad participation of pseudogenes and great clinical value in diseases.

Actually, pseudogenes have strong potential to serve as key regulators in certain diseases, as based on recent studies presented in the tables above due to the following reasons. 1) Wide involvement in diseases: Current studies demonstrate broad pseudogene participation in the pathogenesis and pathology of diseases involving nearly every system and organ of the human body. 2) Close correlations with cancer: The majority of pseudogene studies, nearly 80%, concentrate on the relationship between pseudogenes and cancer, and multiple cancers, such as GBM, BC, HCC, GC, CRC and RCC, are induced by disorders caused by pseudogene expression or function. 3) Prognosis-related expression patterns: A number of pseudogenes have been clinically proven to be expressed in a specific manner for predicting the prognosis of patients with several diseases, indicating their involvement in disease. 4) Effects on cellular biology: Pseudogenes can have a global effect on cellular behavior, including the cell cycle, apoptosis, proliferation, migration, invasion, metabolism, drug resistance and radiotherapy resistance. In addition, the cell microenvironment and angiogenesis can be influenced by pseudogenes. 5) A series of functions in disease modulation: Of note, the ceRNA function of pseudogenes in disease regulation is dominant. Simultaneously, pseudogenes serving as a scaffold to interact with RBPs, triggering specific epigenetic modifications, e.g., DNA methylation and initiating multiple signaling pathways such as PI3K/Akt, Wnt, EMT, JAK/STAT and MAPK have been reported. 6) A single pseudogene in multiple disease regulation: Intriguingly, several pseudogenes can regulate and correlate closely with the diagnosis and prognosis of more than a single disease, e.g., *PTENP1* in HCC, BC, GC, ccRCC and AD, *SUMO1P3* in HCC, GC, CRC and PDAC, and *HMGA1P6/7* in EEC, PT, TC, BC, UCS and OCS. Notably, an increasing number of studies have gradually demonstrated an irreplaceable and connectable role of pseudogenes in the development and progression of diseases.

## Clinical Perspective: A Promising Biotarget

### Diagnosis: A pseudogene is a potential biomarker for disease diagnosis

To alleviate the morbidity and mortality of diseases, especially of several lesions that are typically asymptomatic at the early stage, as well as some lesions that seriously develop rapidly, sensitive biomarkers are of great significance and urgency for diagnosis to create an optimized therapeutic time window for patients. Because of the tremendous efforts from researchers, in particular the emergence of high-throughput sequencing analysis in pseudogene studies, thousands of pseudogenes have been identified and found to play roles in the etiology and pathology of certain diseases. Accordingly, these pseudogenes are likely to be regarded as diagnostic markers. Of note, pseudogenes intrinsically exhibit some characteristics that are helpful in disease diagnosis. 1) Broad-spectrum distribution: Pseudogenes can be detected in a variety of organisms, as well as organs, tissues and blood 30, which is beneficial for acquiring and enhancing the diversity and objectivity of diagnostic indexes for specific diseases. 2) Disease subtype-unique expression: Pseudogenes can differentiate one disease subtype from another because some pseudogenes are only expressed in a single disease subtype 9; thus, they act as a specific disease signature to assist in diagnosis. 3) Tissue-specific expression: Pseudogenes can be applied to distinguish normal tissues from lesion tissues by differential expression, indicating their great power in disease diagnosis and differential diagnosis 10, 30. Intriguingly, the expression profile of multiple pseudogenes in a group can be recognized as a signature with a diagnostic value 136. 4) Dissimilar counterpart expression: In some cases, the expression level of RNA transcript of a pseudogene is not always parallel to its counterpart but is significant for differentiation, especially in several species that only harbor the RNA transcript of the pseudogene 137. 5) Evolutionary conservation: Regardless of the evolutionary distance, pseudogenes are highly conserved, providing an advantageous condition for its measurement and evidence to investigate the probable mechanisms of some zoonoses. In summary, with proper and reasonable development and utilization, these five properties are likely to allow pseudogenes to serve as sensitive and promising biomarkers in disease diagnosis in the future.

### Prognosis: A pseudogene is a possible indicator of life expectancy

Consistent with diagnosis, prognosis is an equally critical aspect that should be considered in the clinic because it determines the best choice of therapy as well as the life expectancy of patients. As a potential biomarker for disease diagnosis, pseudogenes are being proven to be valuable for prognosis 138. The expression level of a single pseudogene can be regarded as helpful in evaluating overall survival (OS). For example, ccRCC patients with low levels of *PTENP1* show a shorter OS rate than do those with high *PTENP1* levels 107; overexpression of *OCT4-pg1* in GC due to aberrant amplification can result in a poor rate of life expectancy in GC patients 105. In addition, SNPs in the pseudogene sequence correlate with prognosis; e.g., HCC patients who carry the GG allele of *E2F3P1* show a worse OS than do those carrying the GA/AA allele 39. Moreover, by combining pseudogene expression with the tumor grade or stage, pseudogenes can indirectly predict OS in several diseases, especially cancer. Intriguingly, a certain number of pseudogenes can be combined as a signature that helps stratify disease risk, as do the clues provided by research in kidney renal clear cell carcinoma (KIRC) 136. Furthermore, recent studies have indicated that pseudogenes are more amenable for determining the prognosis of several diseases than are traditional signatures, such as mRNAs, miRNAs and epigenetic modifications 18, thus elevating the prognostic value of pseudogenes to the next level. Taken together, pseudogenes exhibit enormous potential as effective prognosis indicators of diseases. Future investigations should focus on finding more prognosis-related pseudogenes and using a combination of pseudogenes and traditional signatures or a combination of multiple pseudogenes as signatures to increase efficiency and accuracy in clinical applications.

### Therapeutics: A pseudogene is a promising approach for therapeutic strategy

Although substantial therapeutic methods, especially drugs, are rapidly emerging in recent years, several issues remain that impair their efficiency, such as off-target effects and drug resistance. New and effective biotargets are needed to overcome these obstacles. Taking the features of pseudogenes into account, it is feasible to exploit pseudogenes to develop therapeutic strategies in a novel field for the following reasons. First, a pseudogene is an ideal target for biological treatment. Because they widely expressed in a series of organisms and different specimens, pseudogenes are reported to be involved in multiple processes, including physiology and pathology, are important for homeostasis. Therefore, aberrantly expressed pseudogenes can be used as biological targets for treatment. Second, pseudogenes are a resource for therapy. By sequestering target miRNAs through MREs, pseudogenes can abolish the pathogenic effects of miRNAs or elements with MREs, e.g., RBPs. Additionally, pseudogenes can restrain the pathogenic effects of their counterparts by producing antisense RNAs or siRNAs. Notably, pseudogenes can generate to several lncRNAs that probably exert “lncRNA” functions in disease prevention. Hence, developing and optimizing sequences to create more pseudogene analogs that better facilitate the miRNA “decoy”, along with the antisense RNA, siRNA and lncRNA “producer” functions, may be an ideal strategy. Third, Transcribed pseudogenes can be used as vaccines. One study has shown that peptides from transcribed pseudogenes can be recognized as “antigens” that activate the innate or adaptive immune response in the human body 83. Based on this evidence, disease-specific transcribed pseudogenes can be redesigned to produce peptides with high antigenicity and immunogenicity as well as low toxicity to induce a strong and rapid immune response. In conclusion, pseudogenes provide a novel method and strategy for enriching treatments for certain diseases, especially those that are not sensitive to or resist traditional therapies in the clinic.

## Detection: An Integrated Method

With detection based on DNA sequencing analysis of the whole human genome by GENCODE, over 60,000 total genes have been discovered. However, because of the high similarity in DNA sequences, it is challenging to distinguish pseudogenes from parental genes at the DNA level. Recently, multiple pipelines have been developed and applied to measure pseudogene DNA, such as PseudoPipe 139, PseudoFinder and RetroFinder 140, which markedly enhance the detection efficiency and accuracy of pseudogene DNA. Two comprehensive databases, Encyclopedia of DNA Elements (ENCODE) 141 and Functional Annotation of Mammals (FANTOM) 142, which were built on integration of a series of public pipelines, are currently considered gold standards for pseudogene DNA detection. In fact, as detection methods are constantly being developed, the number of newly found pseudogenes in the genomes of different species will continue to increase.

For pseudogene RNA detection, RNA sequencing analysis is regarded as the first choice that provides precise and thorough detection of all pseudogenes, from subtype to quantity, in a transcriptome, thus creating a plethora of novel information for further exploration of the known and unknown aspects of pseudogene. Moreover, with the rapid development of bioinformatics pipelines, a large number of pseudogene RNA transcripts have been found in different conditions, especially in cancer 136, 143, 144. Microarrays and quantitative real-time polymerase chain reaction (qRT-PCR) are two general methods that have higher sensitivities and specificities but lower costs than RNA sequencing. Nevertheless, extreme care should be taken to ensure that the probes used for microarrays and the primers used for qRT-PCR are specific enough to avoid amplification of parental genes or nonspecific templates. Notably, northern blotting has been utilized for measuring transcribed pseudogenes 29. Furthermore, *in situ* hybridization assays, such as ISH and FISH, have an advantage for investigating the spatiotemporal distribution of pseudogene RNA transcripts. To better understand the intrinsic relationship between a pseudogene and its parental gene or to separate these two factors, gene-editing technology, e.g., the clustered regularly interspaced short palindromic repeats-cas9 (CRISPR-Cas9) system, can be employed to knockout the parental gene without an off-target effect. Additionally, the protein expression levels assessed via western blotting can directly reflect the functional RNA transcripts of pseudogenes at the translational level. Because each method has both advantages and disadvantages in pseudogene DNA or RNA detection, the combined use of multiple methods to acquire a more thorough and accurate result should be the trend, as opposed to a single strategy, in future pseudogene studies.

## Limitations, Challenges and Perspectives

It has been confirmed that the majority of DNA sequences in the human genome do not encode proteins and that the genome is partially composed of pseudogenes. Without a coding function, pseudogenes have been regarded as “junk DNA” or “genomic fossils” for a long period of time, and substantial efforts have been made into devising strategies to eliminate pseudogenes when its parental coding gene was being analyzed 5, 145. However, the real nature of the pseudogene is far more than “trash” or “nonfunctional”. Indeed, recent studies have gradually shed light on the function and involvement of pseudogenes in physiological and pathological conditions. Pseudogenes serve as a function-combined machine that plays different roles at the DNA, RNA or protein level, with participation in the gene regulation network at the transcriptional and posttranscriptional levels. Although pseudogenes are evolutionally conserved, they act as a gene reservoir that effectively allows the genome to carry out novel functions. In addition, a small number of pseudogenes are able to encode proteins, and this finding questions the reasonability of the “pseudo” prefix that conceals the real functions of pseudogenes. “Pseudogene” is a term relative to the parental gene, suggesting sequential variance rather than function. Furthermore, with accumulating evidence confirming pseudogene functions, it is appropriate to create novel nomenclature to better reflect the intrinsic property of these sequences.

Nonetheless, some limitations exist in recent pseudogene studies that future works should focus on. 1) A unified and standard rule for pseudogene naming is needed. In fact, the current pseudogene naming system is relatively chaotic and does not have a unified principle, which is likely to generate errors in gene annotation, especially during sequencing analysis. 2) A certain number of pseudogene investigations have only revealed the differential expression of a single or cluster of pseudogenes in several diseases, whereas their functions and mechanisms have not been fully discussed. 3) The majority of current pseudogene studies concentrate on the correlation between pseudogenes and cancer, and a shortage of evidence on other diseases is notable in this field. 4) Although a substantial number of pseudogenes have been reported to be involved in some diseases, only a small fraction of them meet the requirements of a diagnostic marker or therapy target. Moreover, more *in vitro* experiments, *in vivo* experiments and clinical trials are needed for potential markers to demonstrate their value in the clinic. 5) More attempts should be made to elucidate other pseudogene functions involved in the development and progression of diseases, as opposed to ceRNA behaviors. 6) Additionally, more focus on the regulatory relationships between pseudogenes and other genes in the genome without a restriction to parental genes is needed. 7) Although some studies have reported a few clues regarding the role of pseudogenes in epigenetic modifications, in particular DNA methylation 61, 62, multiple questions remain for other regulation patterns, such as histone modification and chromatin remodeling. Therefore, pseudogene investigations are still in their infancy, and knowledge should be increased.

In addition to these limitations, pseudogene studies are inevitably confronted with the following challenges. First, it is difficult to identify a cluster of pseudogenes with no protein-coding ability because this type of pseudogene cannot be traced via its RNA or protein products. Second, due to the high sequence similarity between a pseudogene and its parental gene, highly specific primers and antibodies that can effectively differentiate them are urgently needed to help with detection. Third, accurately evaluating the outcomes of high-throughput sequencing analysis and matching the data to pseudogene locations hinders their use, especially without a uniform naming principle. Fourth, more precise methods should be introduced for pseudogene studies, such as luciferase reporter assays, northern blotting and CRISPR-Cas9 technology, as qRT-PCR has poor specificity because of the high sequence homology. Furthermore, bioinformatics analyses of pseudogenes, particularly those that can predict the clinical value of pseudogenes, are lacking. Developing databases that are more clinically applicable is of significance for further pseudogene investigations. In this case, many obstacles to pseudogene studies still need to be addressed in the future.

Individualized medicine, which is mainly based on the theory that a specific treatment for a certain patient should be formulated in accordance with his or her own detailed information contained in the genome and epigenome, is critical to improve the therapeutic efficiency for some diseases of unknown etiology and that are not sensitive to or are resistant to traditional therapy, especially cancer. Moreover, with the rapid emergence of high-throughput sequencing technologies, including multiple omics studies, the precision of disease subtype classification and evidence for the utilization of drugs or drug combinations, which relies on variation in gene profiles, has greatly increased. Notably, in this scenario, sensitive biomarkers for disease diagnosis and treatment appear to be crucial because substantial biotargets can be obtained at one time. In fact, pseudogenes are highly valuable in disease diagnosis and prognosis due to their own expression patterns; in some cases, pseudogenes even display higher power than do mRNAs and miRNAs 136, suggesting that pseudogenes are quite appropriate as biomarkers. Additionally, pseudogenes have great potential to be translated into feasible therapeutic targets because of their various properties, particularly ceRNA functions. In summary, although our current knowledge on pseudogenes is preliminary, it is expected that pseudogenes may help to establish novel strategies for clinical applications with regard to disease diagnosis, prognosis and therapeutics, thus accelerating the development of individualized medicine.

## Conclusions

(1) Pseudogenes should no longer be regarded as “junk” or “relics” because multiple functions of pseudogenes at the DNA, RNA and protein levels have recently been discovered, especially their ability to encode functional proteins, which seriously contradicts their previous “nonfunctional” identification. Herein, we shed light on the details of their functions and roles in gene regulation at the molecular level.

(2) Pseudogenes are a product of gene mutation and serve as a gene “repository” and can generally be classified into three categories according to distinct biogenesis processes, storing and expanding genetic information.

(3) Pseudogenes possess a series of clinic-associated features, including wide and uneven distribution, spatiotemporal and unique expression patterns and evolutionary conservation, which allow them to potentially be involved in disease diagnosis, prognosis and therapeutics as promising biomarkers or biotargets, suggesting their high value in clinical applications, even in individualized medicine.

(4) Current pseudogene detection methods have been largely improved at the DNA and RNA levels, in particular with the emergence of high-throughput sequencing analysis. However, pseudogene studies are still in their infancy and affected by various limitations and challenges that need further investigation.

## Figures and Tables

**Table 1:** Pseudogenes widely participate in the pathogenesis of different diseases

Diseases	Pseudogenes	Species	Tissues	Sponge Targets	Expression Pattern	Overall Survival	Potential Functions / Applications	References
HCC	*ANXA2P2*	Homo Sapiens	Liver	-	Upregulated	Decline	Induces migration and invasion of HCC cells *in vitro*	Wang et al. (2019) 84
	*RACGAP1P*	Homo Sapiens	Cell Line	miR-15-5p	Upregulated	Decline	Sequestrates miR-15-5p from *RACGAP1*; Activates RhoA/ERK signaling	Wang et al. (2019) 85
	*RP11-564D11.3*	Homo Sapiens	Liver	miR-9-5p; miR-101-3p; miR-200 family	Upregulated	Decline	Sequestrates miR-9-5p, miR-101-3p, and miR-200 family to target *VEGFA* to initiate and promote HCC	Song et al. (2019) 86
	*SUMO1P3*	Homo Sapiens	Liver; Cell Line	-	Upregulated	Decline	Promotes cell proliferation, migration, invasion and radiation resistant of HCC *in vitro*	Zhou et al. (2019) 87
	*UBE2CP3*	Homo Sapiens	Liver; Cell Line	-	Upregulated	Decline	Induces angiogenesis functions of HUVECs by activating ERK/HIF-1α/p70S6K/VEGFA signaling	Lin et al. (2018) 88
	*OCT4-pg1*	Homo Sapiens	Liver; Cell Line	-	Upregulated	-	Promotes proliferation of HCC cells by inducing activation of AKT	Pan et al. (2018) 89
	*OCT4-pg4*	Homo Sapiens	Liver; Cell Line	miR-145	Upregulated	Decline	Serves as a miR-145 decoy to increase its parental gene *OCT4* expression to promote HCC	Wang et al. (2013) 74
	*PTENP1*	Homo Sapiens	Liver; Cell Line	miR-193a-3p	Downregulated	Elevation	Sequestrates miR-193a-3p to suppress cell growth, migration and invasion of HCC via PTEN pathway	Qian et al. (2017) 90
	*UBE2CP3*	Homo Sapiens	Liver; Cell Line	-	Upregulated	Decline	Promotes epithelial to mesenchymal transition (EMT) *in vitro* and *in vivo*	Cao et al. (2017) 91
	*INTS6P1*	Homo Sapiens	Liver; Cell Line	miR-17-5p	Downregulated	-	Serves as a miR-17-5p decoy to induce its parental gene *INTS6* expression to inhibit HCC	Peng et al. (2015) 92
	*E2F3P1*	Homo Sapiens	Liver	-	-	-	The A allele of rs9909601 in *E3F3P1* is positively correlated with a better prognosis than the G allele	Pan et al. (2014) 39 Liu et al. (2013) 93
BC	*PTTG3P*	Homo Sapiens	Breast	miR-129-5p; miR-376c-3p; miR-383-5p	Upregulated	Decline	Serves as a miRNA decoy to exert an oncogenic role via a miRNA-mRNA regulatory network	Lou et al. (2019) 94
	*PTENP1*	Homo Sapiens	Breast	-	Downregulated	-	Inhibits growth and migration and enhances doxorubicin sensitivity in ER-negative cells; Increases ER-positive cell growth and decreases ERα	Yndestad et al. (2018) 95
	*CKS1BP7*	Homo Sapiens	Breast	-	Upregulated	-	Interacts with *IGF1R* to enhance cell proliferation	Liu et al. (2018) 96
	*CYP4Z2P*	Homo Sapiens	Breast; Cell Line	miR-125a-3p	Upregulated	-	Sequesters miR-125a-3p to trigger a hTERT-dependent apoptotic inhibition; Induces phosphorylation of ERK1/2 and PI3K/Akt to enhance tumor-angiogenesis	Li et al. (2017) 97 Zheng et al. (2015) 98 Zheng et al. (2014) 99
	*HMGA1P7*	Homo Sapiens	Breast Cell Line	miR-15; miR-16; miR-214; miR-761	Upregulated	-	Acts as a ceRNA to promote *H19* and *Igf2* expression to induce MCF-7 cell proliferation *in vitro*	De Martino et al. (2016) 100
GC	*NANOGP8*	Homo Sapiens	Cell Line	-	Upregulated	-	Binds to the promoter region of DBC1 to induce cell proliferation and suppress apoptosis	Li et al. (2019) 101
	*GBAP1*	Homo Sapiens	Stomach	miR-212-3p	-	-	Serves as a miR-212-3p decoy to induce *GBA*; SNP rs2990245 in *GBAP1* influences the DNA methylation status of its own promoter region	Ma et al. (2019) 102
	*PTENP1*	Homo Sapiens	Stomach; Cell Line	-	Downregulated	-	G allele of rs7853346 in *PTENP1* is negatively correlated with the risk of GC; Suppresses GC proliferation, migration and invasion	Ge et al. (2017) 103 Guo et al. (2016) 104
	*OCT4-pg1*	Homo Sapiens	Stomach; Cell Line	-	Upregulated	Decline	Elevates expression of various growth factors to enhance GC cell proliferation and angiogenesis	Hayashi et al. (2015) 105
	*SUMO1P3*	Homo Sapiens	Stomach	-	Upregulated	Decline	Positively correlates with GC growth, differentiation and lymphatic metastasis	Mei et al. (2013) 10
RCC	*DUXAP8*	Homo Sapiens	Kidney; Cell Line	-	Upregulated	Decline	Alters miR-126/*CED-9* signaling to promote RCC cell proliferation and migration	Huang et al. (2018) 106
ccRCC	*PTENP1*	Homo Sapiens	Kidney; Cell Line	miR-21	Downregulated	Elevation	Serves as a miR-21 decoy to increase *PTEN*; Inhibits cell growth, migration, invasion and sensitivity to cisplatin and gemcitabine	Yu et al. (2014) 107
LUAD	*CSDAP1*	Homo Sapiens	Lung; Cell Line	-	Upregulated	Decline	Positively correlates with LUAD occurrence, development and prognosis	Xu et al. (2018) 108
	*CHIAP2*	Homo Sapiens	Lung; Cell Line	miR-873-3p; miR-3614-5p	Downregulated	-	Serves as a miRNA decoy to regulate NFATC2 or GSK3β in WNT signaling pathway	Shang et al. (2019) 109
NSCLC	*DUXAP8*	Homo Sapiens	Lung; Cell Line	-	Upregulated	Decline	Recruits LSD1 and EZH2 to the promoters of *EGR1* and *RHOB* to enhance NSCLC malignant phenotypes	Sun et al. (2017) 110
	*DUXAP10*	Homo Sapiens	Lung; Cell Line	-	Upregulated	Decline	Interacts with LSD1 to epigenetically silence *LATS2* and *RRAD* to induce tumorigenesis in NSCLC	Wei et al. (2017) 111
CRC	*MYLKP1*	Homo Sapiens	Colon; Cell Line	-	Upregulated	-	Suppresses its parental gene *MYLK* expression; Two SNPs, rs12490683 and rs12497343 in *MYLKP1* significantly elevate the risk of CRC	Lynn et al. (2018) 112 Han et al. (2011) 80
	*SUMO1P3*	Homo Sapiens	Colon; Cell Line	-	Upregulated	Decline	Induces expression of *cyclin D1*, *Vimentin* and *VEGFA* and decreases *E-cadherin* to promote tumorigenesis and angiogenesis of CRC	Zhang et al. (2017) 113
	*TPTE2P1*	Homo Sapiens	Colon; Cell Line	-	Upregulated	Decline	Induces cell cycle progression and inhibits apoptosis of CRC cells *in vitro* and *in vivo*	Dai et al. (2019) 114
EEC	*HMGA1P6 HMGA1P7*	Homo Sapiens	Endometrium	-	Upregulated	Decline	Positively correlates with expression of its parental gene* HMGA1* and EEC progression	Palumbo Junior et al. (2019) 115
UCS	*HMGA1P6*	Homo Sapiens	Uterus	let-7a; miR-26a; miR-16; miR-214	Upregulated	-	Serves as a miRNA decoy implicated in downregulation of the *HMGA1*-targeting miRNAs, contributing to *HMGA1* overexpression	Brunetti et al. (2019) 116
OCS	*HMGA1P6 HMGA1P7*	Homo Sapiens	Ovary	let-7a; miR-26a; miR-16; miR-214	Upregulated	-	Serves as miRNA decoys implicated in downregulation of the *HMGA1*-targeting miRNAs, contributing to *HMGA1* overexpression	Esposito et al. (2014) 72 Brunetti et al. (2019) 116
GBM	*DUXAP8*	Homo Sapiens	Brain; Cell Line	-	Upregulated	Decline	Promotes cell proliferation and colony formation of GBM *in vitro*	Zhao et al. (2019) 117
	*LGMNP1*	Homo Sapiens	Cell Line	-	Upregulated	-	Reduces DNA damage processes and apoptosis to help acquire radiotherapy resistance	Xu et al. (2019) 118
PT	*HMGA1P6 HMGA1P7*	Homo Sapiens	Pituitary; Cell Line	-	Upregulated	-	Serve as ceRNAs to induce parental gene *HMGA1* expression and increase cell proliferation and migration	Esposito et al. (2015) 119
CC	*OCT4-pg1*	Homo Sapiens	Cervix; Cell Line	-	Upregulated	-	Induces cell proliferation and migration and inhibits apoptosis of CC cells *in vitro* and *in vivo*	Yu et al. (2019) 120
PDAC	*DUXAP8*	Homo Sapiens	Pancreas; Cell Line	-	Upregulated	Decline	Serves as a scaffold for EZH2 and LSD1 to epigenetically silence *CDKN1A* and *KLF2*	Lian et al. (2018) 121
	*SUMO1P3*	Homo Sapiens	Pancreas; Cell Line	-	Upregulated	Decline	Induces development of EMT to enhance cell proliferation, migration and invasion *in vitro*	Tian et al. (2018) 122
ESCC	*TUSC2P*	Homo Sapiens	Esophagus; Cell Line	miR-17-5p; miR-520a-3p; miR-608; miR-611	Downregulated	Elevation	Plays a tumor suppressive role in a miRNA-binding manner to induce a cell growth and invasion inhibition	Liu et al. (2018) 123
	*FTH1P3*	Homo Sapiens	Esophagus; Cell Line	-	Upregulated	-	Enhances cell proliferation, migration, and invasion by activating Sp1 and NF-κB	Yang et al. (2018) 124
DLBCL	*Braf-rs1*	Murine	Spleen; Cell Line	miR-134; miR-543; miR-653	Upregulated	Decline	Functions in a ceRNA manner to elevate *BRAF* expression and to regulate MAPK signaling and cell proliferation *in vitro* and *in vivo*	Karreth et al. (2015) 73
	*BRAFP1*	Homo Sapiens	Spleen; Cell Line	miR-30a; miR-182; miR-876; miR-590	Upregulated	Decline	Functions in a ceRNA manner to elevate *BRAF* expression and to regulate MAPK signaling and cell proliferation *in vitro* and *in vivo*	Karreth et al. (2015) 73
AML	*OCT4-pg1*	Homo Sapiens	Blood	-	Downregulated	Elevation	Contributes to the diagnosis and prognosis of AML	Yi et al. (2019) 125
CML	*OCT4-pg1*	Homo Sapiens	Cell Line	-	-	-	Interacts directly with OCT-4, SOX2, and NANOG and indirectly with ABC transporters	Lettnin et al. (2019) 126
TC	*HMGA1P6 HMGA1P7*	Homo Sapiens	Thyroid; Cell Line	miR-15; miR-16; miR-214; miR-761	Upregulated	-	Serve as miRNA decoys to induce parental gene *HMGA1* and cancer-related genes expression to promote malignant phenotypes of cells *in vitro*	Esposito et al. (2014) 72
OSCC	*FTH1P3*	Homo Sapiens	Oral; Cell Line	-	Upregulated	Decline	Promotes cell proliferation, migration and invasion by inducing PI3K/Akt/GSK3β/Wnt/β-catenin signaling	Liu et al. (2018) 127
BA	*ANXA2P3*	Homo Sapiens	Liver; Cell Line	-	Upregulated	-	Induces cell cycle progression and growth but inhibits apoptosis by activating *ANXA2*/*ANXA2P3* signaling	Nuerzhati et al. (2019) 128
SPE	*HK2P1*	Homo Sapiens	Decidua; Cell Line	miR-6887-3p	Downregulated	-	Promotes glycolytic metabolism, and HESC decidualization by sequestering miR-6887-3p	Lv et al. (2018) 129
	*PGK1P2*	Homo Sapiens	Decidua	miR-330-5p	Downregulated	-	Sequesters miR-330-5p to affect decidualization by regulating angiogenesis and glycolysis metabolism	Tong et al. (2018) 130
Schizophrenia	*NDUFV2P1*	Homo Sapiens	Brain; Cell Line	-	Upregulated	-	Negatively correlates with pre- and mature *NDUFV2* and with CoI-driven cellular respiration	Bergman et al. (2018) 131
ASD	*lncLRFN5-10*	Homo Sapiens	Cell Line	-	Downregulated	-	Positively affects expression of non-deleted *LRFN5*	Cappuccio et al. (2019) 132
OA	*PMS2L2*	Homo Sapiens	Cell Line	miR-203	Downregulated	-	Serves as a miR-203 decoy to block Wnt/β-catenin and JAK/STAT signaling pathway	Li et al. (2019) 133
AD	*PTENP1*	Homo Sapiens	Aorta; Cell Line	miR-21	Upregulated	-	Induces *PTEN* to promote apoptosis and to inhibit HASMC growth by competing for miR-21	Lai et al. (2019) 134
MM	*PDIA3P*	Homo Sapiens	Bone Marrow; Cell Line	-	Upregulated	Decline	Interacts with c-Myc to bind to G6PD promoter and to induce PPP flux to affect cell growth and drug resistant	Yang et al. (2018) 135

***Abbreviations:*** AD: aortic dissection; AML: acute myelocytic leukemia; ASD: autism spectrum disorder; BA: biliary atresia; BC: breast cancer; CC: cervical cancer; ccRCC: clear cell renal cell carcinoma; ceRNA: competing endogenous RNA; CML: chronic myelocytic leukemia; CRC: colorectal cancer; DLBCL: diffuse large B cell lymphoma; EEC: endometrioid endometrial carcinomas; EMT: epithelial to mesenchymal transition; ESCC: esophageal squamous cell carcinoma; GBM: glioblastoma; GC: gastric cancer; HASMC: human aortic smooth muscle cell; HCC: hepatocellular carcinoma; HESC: human endometrial stromal cell; HUVEC: human umbilical vein endothelial cell; LUAD: lung adenocarcinoma; miRNA: microRNA; MM: multiple myeloma; mRNA: messenger RNA; NSCLC: non-small cell lung cancer; OA: osteoarthritis; OCS: ovarian carcinosarcomas; OSCC: oral squamous cell carcinoma; PDAC: pancreatic ductal adenocarcinoma; PT: pituitary tumor; RCC: renal cell carcinoma; SNP: single nucleotide polymorphism; SPE: severe preeclampsia; TC: thyroid carcinoma; UCS: uterine carcinosarcomas.

**Table 2:** Overexpression or knockdown effects of pseudogenes *in vitro* and *in vivo*

Pseudogenes	Treatments	Diseases	Effects	References
*ANXA2P2*	Knockdown	HCC	Inhibits migration and invasion of HCC cells *in vitro*	Wang et al. (2019) 84
*RACGAP1P*	Overexpression	HCC	Sequestrates miR-15-5p to trigger RhoA/ERK signaling; Promotes cell growth and migration of HCC *in vitro* and *in vivo*	Wang et al. (2019) 85
*SUMO1P3*	Knockdown	HCC	Suppresses proliferation, migration, invasion and enhances radio-sensitivity of HCC cells	Zhou et al. (2019) 87
*UBE2CP3*	Overexpression	HCC	Enhances HUVEC proliferation, migration and tube formation by inducing ERK/HIF-1a/p70S6K/VEGFA signaling	Lin et al. (2018) 88
*OCT4-pg1*	Overexpression	HCC	Promotes HCC cell proliferation by activating AKT *in vitro*	Pan et al. (2018) 89
*OCT4-pg4*	Knockdown	HCC	Suppresses cell proliferation and colony formation *in vitro* and *in vivo*	Wang et al. (2013) 74
*PTENP1*	Overexpression	HCC	Inhibits HCC growth, migration and invasion via the *PTEN* pathway *in vitro* and *in vivo*	Qian et al. (2017) 90
*UBE2CP3*	Knockdown	HCC	Suppresses the epithelial to mesenchymal transition *in vitro* and *in vivo*	Cao et al. (2017) 91
*INTS6P1*	Knockdown	HCC	Induces HCC growth and migration *in vitro* and *in vivo*	Peng et al. (2015) 92
*PTENP1*	Overexpression	BC	Promotes ER-positive cell growth and decreases *PTEN1* and ERα; Inhibits growth and migration and enhances *PTEN1* in ER-negative cells	Yndestad et al. (2018) 95
*CYP4Z2P*	Knockdown	BC	Releases miR-125a-3p to inhibit a hTERT-mediated apoptotic repression	Li et al. (2017) 97
*NANOGP8*	Knockdown	GC	Suppresses DBC1 to inhibit cell proliferation and promote apoptosis	Li et al. (2019) 101
*OCT4-pg1*	Overexpression	GC	Promotes expression levels of different growth factors to induce GC cell proliferation and angiogenesis *in vitro* and *in vivo*	Hayashi et al. (2015) 105
*DUXAP8*	Knockdown	RCC	Increases miR-126 to inhibit *CED-9* expression to suppress RCC cell growth and migration *in vitro*	Huang et al. (2018) 106
*PTENP1*	Overexpression	ccRCC	Sequesters miR-21 to induce *PTEN* expression to suppress cell growth, migration, and invasion and increase sensitivity to cisplatin and gemcitabine *in vitro* and *in vivo*	Yu et al. (2014) 107
*CHIAP2*	Overexpression	LUAD	Sequesters miR-873-3p and miR-3614-5p to inhibit cell proliferation and invasion	Shang et al. (2019) 109
*DUXAP8*	Knockdown	NSCLC	Inhibits recruitment of LSD1 and EZH2 to the promoter regions of *EGR1* and *RHOB* to suppress NSCLC malignant phenotypes *in vitro* and *in vivo*	Sun et al. (2017) 110
*DUXAP10*	Knockdown	NSCLC	Promotes transcriptions of *LATS2* and *RRAD* to restrain NSCLC cell growth, migration and invasion *in vitro* and *in vivo*	Wei et al. (2017) 111
*MYLKP1*	Overexpression	CRC	Suppresses expression of *MYLK* and promotes cell proliferation and migration	Lynn et al. (2018) 112 Han et al. (2011) 80
*SUMO1P3*	Knockdown	CRC	Suppresses expression levels of *cyclin D1*, *Vimentin*, and *VEGFA* and enhances *E-cadherin* to reduce CRC malignant behavior *in vitro* and *in vivo*	Zhang et al. (2017) 113
*TPTE2P1*	Knockdown	CRC	Promotes cell cycle arrest at S phase and induces apoptosis by activating the *BCL2*/*Caspase 3* signaling cascade *in vitro* and *in vivo*	Dai et al. (2019) 114
*DUXAP8*	Knockdown	GBM	Suppresses cell proliferation and colony formation in GBM *in vitro*	Zhao et al. (2019) 117
*LGMNP1*	Overexpression	GBM	Reduces DNA damage processes and apoptosis to resist radiotherapy	Xu et al. (2019) 118
*HMGA1P6 HMGA1P7*	Overexpression	PT	Functions as a decoy for *HMGA1*-targeting miRNAs to promote *HMGA1* expression and induce AtT20 cell proliferation and migration	Esposito et al. (2015) 119
*OCT4-pg1*	Knockdown	CC	Suppresses cell proliferation and migration and promotes apoptosis of CC cells *in vitro* and *in vivo*	Yu et al. (2019) 120
*DUXAP8*	Knockdown	PDAC	Increases expression levels of *CDKN1A*, and *KLF2* to inhibit cell proliferation and promote apoptosis *in vitro* and *in vivo*	Lian et al. (2018) 121
*SUMO1P3*	Knockdown	PDAC	Reverses the process of EMT to inhibit cell proliferation, migration and invasion *in vitro*	Tian et al. (2018) 122
*TUSC2P*	Overexpression	ESCC	Suppresses cell growth and invasion in a miRNA-binding manner *in vitro*	Liu et al. (2018) 123
*FTH1P3*	Knockdown	ESCC	Inhibits cell growth, migration and invasion by silencing Sp1 and NF-κB *in vitro*	Yang et al. (2018) 124
*Braf-rs1*	Overexpression	DLBCL	Sequesters miRNAs to activate MAPK signaling and cell proliferation *in vitro* and *in vivo*	Karreth et al. (2015) 73
*BRAFP1*	Overexpression	DLBCL	Sequesters miRNAs to activate MAPK signaling and cell proliferation *in vitro* and *in vivo*	Karreth et al. (2015) 73
*OCT4-pg1*	Knockdown	CML	Reduces expression and activity of *OCT4* as well as *ABCB1* and activates *ALOX5* and *ABCC1* to render cell sensitive to chemotherapy	Lettnin et al. (2019) 126
*HMGA1P6 HMGA1P7*	Overexpression	TC	Sequesters HMGA1-targeting miRNAs to increase cell proliferation, migration and invasion and suppress apoptosis *in vitro*	Esposito et al. (2014) 72
*FTH1P3*	Knockdown	OSCC	Suppresses cell proliferation, migration and invasion by reducing activation of PI3K/Akt/GSK3β/Wnt/β-catenin signaling	Liu et al. (2018) 127
*ANXA2P3*	Knockdown	BA	Suppresses proliferation, increases apoptosis and induces cell cycle arrest in G1 phase *in vitro* by inhibiting *ANXA2*/*ANXA2P3* signaling	Nuerzhati et al. (2019) 128
*HK2P1*	Knockdown	SPE	Inhibits glucose uptake, lactate production and decidualization in HESCs by releasing miR-6887-3p	Lv et al. (2018) 129
*PTENP1*	Overexpression	AD	Increases expression of *PTEN* to induce apoptosis and inhibit proliferation of HASMCs by sequestering miR-21 *in vitro* and *in vivo*	Lai et al. (2019) 134

***Abbreviations*:** AD: aortic dissection; BA: biliary atresia; BC: breast cancer; CC: cervical cancer; ccRCC: clear cell renal cell carcinoma; CML: chronic myelocytic leukemia; CRC: colorectal cancer; DLBCL: diffuse large B cell lymphoma; EMT: epithelial to mesenchymal transition; ESCC: esophageal squamous cell carcinoma; GBM: glioblastoma; GC: gastric cancer; HASMC: human aortic smooth muscle cell; HCC: hepatocellular carcinoma; HESC: human endometrial stromal cell; HUVEC: human umbilical vein endothelial cell; LUAD: lung adenocarcinoma; NSCLC: non-small cell lung cancer; OSCC: oral squamous cell carcinoma; PDAC: pancreatic ductal adenocarcinoma; PT: pituitary tumor; RCC: renal cell carcinoma; SPE: severe preeclampsia; TC: thyroid carcinoma.

## Abbreviations

3'-UTR: 3'-untranslated region
ACYL3: O-acyltransferase like, pseudogene
BRAF: B-Raf proto-oncogene, serine/threonine kinase
BRAFP1: BRAF pseudogene 1
BRCA1: BRCA1 DNA repair associated
ccRCC: clear cell renal cell carcinoma
ceRNA: competing endogenous RNA
CNS: central nervous system
CRISPR-Cas9: clustered regularly interspaced short palindromic repeats cas9
DNMT3A: DNA methyltransferase 3 alpha
E2F3: E2F transcription factor 3
ENCODE: Encyclopedia of DNA Elements
EZH2: enhancer of zeste 2 polycomb repressive complex 2 subunit
FANTOM: Functional Annotation of Mammals
FER1L4: fer-1-like family member 4 (pseudogene)
FISH: fluorescence in situ hybridization
FUT2: fucosyltransferase 2
G9A: euchromatic histone lysine methyltransferase 2
GC: gastric cancer
H3K27me3: histone trimethylated at lysine 27
H3K9me3: histone trimethylated at lysine 9
HCC: hepatocellular carcinoma
HP1a: Heterochromatin Protein 1A
HTR7: 5-hydroxytryptamine receptor 7
INTS6: integrator complex subunit 6
INTS6P1: integrator complex subunit 6 pseudogene 1
ISH: *in situ* hybridization
KIRC: kidney renal clear cell carcinoma
KLK4: kallikrein related peptidase 4
KLKP1: kallikrein pseudogene 1
lncRNA: long noncoding RNA
MAPK: mitogen-activated protein kinase
MGA: MAX dimerization protein MGA
miRNA: microRNA
MRE: miRNA response element
MYLK: myosin light chain kinase
MYLKP1: myosin light chain kinase pseudogene 1
NANOG: Nanog homeobox
NANOGP8: Nanog homeobox retrogene P8
NEK8: NIMA related kinase 8
NOS: nitric oxide synthase
OCT4: POU class 5 homeobox 1
ORF: open reading frame
PARP: poly (ADP-ribose) polymerase
PGAM1: phosphoglycerate mutase 1
PGAM3: phosphoglycerate mutase processed gene
PMS2: PMS1 homolog 2, mismatch repair system component
PMS2CL: PMS2 C-terminal-like pseudogene
PPM1K: protein phosphatase, Mg^2+^/Mn^2+^ dependent 1K
PsiBRCA1: BRCA1 pseudogene 1
psiTPTE22: TPTE pseudogene 1
PTEN: phosphatase and tensin homolog
PTENP1: phosphatase and tensin homolog pseudogene 1
PTPN12: protein tyrosine phosphatase non-receptor type 12
qRT-PCR: quantitative reverse transcription polymerase chain reaction
RBP: RNA binding protein
RNAi: RNA interference
SEC1P: secretory blood group 1, pseudogene
siRNA: small interfering RNA
SNP: single-nucleotide polymorphism
SUMO1: small ubiquitin like modifier 1
SUMO1P3: SUMO1 pseudogene 3
SUV39H1: suppressor of variegation 3-9 homolog 1
TPTE: transmembrane phosphatase with tensin homology
